# Treatment of Corneal Spots

**Published:** 1892-10

**Authors:** 


TREATMENT OF CORNEAL SPOTS.—(*Bollettino d'oculistica*, February 15, 1892.) Small dots of opacity of the cornea can be seen in their normal type when the evolution of the repairing process takes place in a natural manner and without the employment of any kind of treatment. When a spot of more or less thickness is formed by the union of the cicatricial tissue of the deposits of conjunctival secretions with the hypertrophied epithelial masses, we can endeavor to restore to the cornea, its transparency, by the transformation of that opacity into a sore or an ulcer. The author of this excellent article, Dr. A. Simi, is of the opinion that preference should be given to caustics to attain the desired result, and of all of them, he chooses iodide of potassium in a concentrated form. He considers it more suitable than citric acid, on account of its dissolving action when applied to calcareous depositions as well as to epithelial masses.

The clinical case reported by the author is entirely conclusive, because the leucoma treated by this method, which had entirely abolished the visual function, was cured without leaving the least trace on the cornea. The *modus faciendi* of the treatment is the following: the applications are practiced every day with a fine brush moistened with the solution, and previously passed over a crystal of citric acid. They are continued until the complete clearing out of the opacity occurs. Strict antisepsis has to be observed during the entire course of this treatment, which is a long one, the healing taking place very slowly, and sometimes it is necessary to prolong it by making new cauterizations.—*Inter. Med. Mag.*

